# SYNTHESIS AND APPLICATION OF QUANTITATIVE METHODS FOR MODELING CHANGE IN MULTIMORBIDITY AND RELATED HEALTH DOMAINS

**DOI:** 10.1093/geroni/igad104.0756

**Published:** 2023-12-21

**Authors:** Nicholas Bishop, Corey Nagel, Ana Quiñones

## Abstract

The longitudinal examination of multimorbidity (≥ 2 chronic conditions) and related health outcomes (e.g., cognition, disability, mortality) has the potential to identify disparities and intervention points in the age-related progression of chronic disease. This symposium will organize discussion of methodological and applied research projects that review and demonstrate the use of longitudinal methods to address critical questions in the study of multimorbidity and related health domains. The lead presentation “Longitudinal Methods for Assessing Progression of Multimorbidity and Related Health Outcomes” will review the use and limitations of several longitudinal methods that can be used to study the progression of multimorbidity and linked health outcomes. Following presentations will demonstrate the application of specific longitudinal methods to key research questions in the study of multimorbidity. “Emerging Cohort Disparities in Multimorbidity in U.S. Adults Entering Early Older Adulthood” presents evidence of cohort disparities in multimorbidity trajectories using mixed-effects regression to estimate age, period, and cohort effects, “Personalized and Typical Concurrent Risk: Joint Models of Recurrent Outcomes and Mortality by Race/Ethnicity” applies joint models of self-rated health, disability, and mortality to examine disparities among minoritized groups, and “Sequence Analysis of Cardiometabolic Multimorbidity and Association With Subsequent Dementia” identifies common patterning of cardiometabolic conditions over a 5-year period and their relationship with prospective dementia classification. This symposium will encourage attendees to apply the presented methods to their own multimorbidity research questions and demonstrate how these methods are being applied to address key gaps in our understating of multimorbidity progression in older adulthood. This is a Measurement, Statistics, and Research Design Interest Group Sponsored Symposium.

